# An Early Warning System for the Differential Diagnosis of In-Hospital Acute Kidney Injury for Better Patient Outcome: Study of a Quality Improvement Initiative

**DOI:** 10.3390/ijerph19063704

**Published:** 2022-03-20

**Authors:** Ming-Ju Wu, Shih-Che Huang, Cheng-Hsu Chen, Ching-Yao Cheng, Shang-Feng Tsai

**Affiliations:** 1 Division of Nephrology, Department of Internal Medicine, Taichung Veterans General Hospital, Taichung 407, Taiwan; wmj530@gmail.com (M.-J.W.); cschen@vghtc.gov.tw (C.-H.C.); 2 Department of Post-Baccalaureate Medicine, College of Medicine, National Chung Hsing University, Taichung 402, Taiwan; 3 Division of Clinical Information, Center of Quality Management, Taichung Veterans General Hospital, Taichung 407, Taiwan; cucu0214@gmail.com; 4 Department of Emergency Medicine, Taichung Veterans General Hospital, Taichung 407, Taiwan; 5 Department of Life Science, Tunghai University, Taichung 407, Taiwan; 6 Department of Pharmacy, Taichung Veterans General Hospital, Taichung 407, Taiwan; chingyao@vghtc.gov.tw; 7 School of Pharmacy, China Medical University, Taichung 404, Taiwan; 8 School of Medicine, National Yang-Ming University, Taipei 112, Taiwan

**Keywords:** early warning system (EWS), acute kidney injury (AKI), electronic health information systems (EHIS), inpatient, diagnosis, outcome

## Abstract

Background: Acute kidney injury (AKI) is a syndrome with heterogeneous causes and mechanisms. An early warning system (EWS) for AKI was created to reduce the incidence and improve outcomes. However, the benefits of AKI-EWS remain debatable. Methods: We launched a project to design and create AKI-EWS for inpatients in our institute. Incidence of AKI and its outcome before and after the implementation of AKI-EWS were collected for analysis. Results: We enlisted a stakeholder map before creating AKI-EWS. We then started an action plan for this initiative. The diagnosis was automatic and based on the definition of Kidney Disease: Improving Global Outcomes (KDIGO). The differential diagnosis of causes of AKI was also automatic. Users are to adjust the threshold of detection. After the implementation of this AKI-EWS, the incidence of AKI fell. The proportion of AKI > 4% was reduced significantly (47.7% and 41.6%, *p* = 0.010) in patients with serum creatinine measured. The proportion of AKI > 0.9% also dropped significantly (51.67% and 35.94%, *p* = 0.024) in all inpatients. Trends of AKI outcomes also showed improvement. The loading of consultation of nephrologists decreased by 15.5%. Conclusions: Through well-designed AKI-EWS, the incidence of AKI dropped, showing improved outcomes. The factors affecting benefits from AKI-EWS included high-risk identification (individual threshold detection), timely and automatic diagnosis, real-time alerting on electronic health information systems, fast self-diagnosing of the cause of AKI, and coverage of all inpatients.

## 1. Introduction

Acute kidney injury (AKI) is characterized by a rise in the serum creatinine level or a declined urine output within days. Patients with AKI have a variety of clinical presentations. The outcome varies, including full recovery, acute kidney disease (AKD), chronic kidney disease (CKD), CKD G5CKD G5, or dialysis and mortality. In a large meta-analysis of 110 studies on AKI (429,535 patients) according to the Kidney Disease: Improving Global Outcomes (KDIGO) definition, the mortality increased to 23% [1]. For hospitalized AKI patients, those discharged have a higher risk of death, rehospitalization, and progressive CKD and CKD G5 [2]. Currently, no global standard is available for the prevention, recognition, treatment, and follow-up of AKI because of the vast differences in care delivery. Recently, a quality improvement projection for AKI (“zero preventable deaths by 2025”) by the International Society of Nephrology’s 0 by 25 initiative (a human rights case for nephrology) was reported [3]. It was aimed to establish awareness and to reduce variations in care delivery for AKI. Early diagnosis of AKI is necessary for timely intervention to improve outcomes. Early nephrologist involvement in hospital-acquired AKI suggested patient benefit [4]. Therefore, early recognition to raise awareness is our first step.

Prompt diagnosis of AKI is important. First, AKI is not associated with specific symptoms and signs. Second, the diagnosis of AKI relies on laboratory measurements (serum creatinine) and urine amount. Usually, missing checkups or serum creatinine measurements, or collection of urine delays diagnosis of AKI. Third, the diagnostic criteria are based on different diagnostic criteria, KDIGO definition and staging system [5,6], RIFLE criteria (risk, injury, failure, loss of kidney function, and end-stage kidney disease) [7], and Acute Kidney Injury Network (AKIN) and others [8,9,10]. The KDIGO guidelines [5,6] define AKI as presenting one of the following conditions: an increase in serum creatinine by ≥0.3 mg/dL within 48 h; an increase in serum creatinine to ≥1.5 times baseline, which is known or presumed to have occurred within the prior seven days; a urine volume of <0.5 mL/kg/h for six hours. In clinical practice, clinicians usually used a staging system of AKI from the KDIGO criteria to evaluate the severity of AKI. It is suggested by the KDIGO guidelines to tailor management to the AKI stage. Staged-based management of AKI can improve the response to therapy according to different severity of AKI [6]. In stage 1 AKI, the clinician should perform a noninvasive diagnostic workup and also consider an invasive diagnostic workup. In stage 2 AKI, the clinician should check for changes in drug dosing and consider renal replacement therapy and ICU admission. Finally, in stage 3 AKI, clinicians should avoid subclavian catheters if possible. Even though the current consensus is that the KDIGO definition of AKI is most favored, memorizing the criteria of KDIGO for AKI is not easy. The complicated criteria of AKI impair the awareness of AKI. Therefore, an automatic early warning system (EWS) for AKI is helpful. However, not all applications of EWS for AKI benefit the AKI outcome because of differences in the data collection system, i.e., with or without intervention [11].

In this study, we created a quality improvement (QI) project to improve the diagnosis and outcome of AKI. QI can improve the quality of processes to improve performance. QI programs are critical because they improve patient outcomes, the efficiency of staff, and reduce waste due to failed processes. This QI focused on the analysis of the current status of AKI in our institute, creation of EWS, interventions of AKI, and feedback for this system. In addition to creating an EWS for AKI, we were also interested in the effect of this notification on AKI incidence and outcome in our institute. We also sought to identify the key point that determined a better outcome of AKI after the implantation of AKI-EWS.

## 2. Material and Methods

### 2.1. Patients and Intervention (EWS)

In this study, we present the whole process of the EWS of AKI. All inpatients enrolled were >20 years old except patients who underwent dialysis. This system has been in working condition since September 2018. The content management system was later created in September 2019. In March 2020, EWS was created in the Electronic Health Information Systems (EHIS) and opened to all users. This EWS will screen all adult inpatients (including all hospital settings except outpatients, such as ordinary ward, intensive care unit, emergent room, and delivery room) every midnight based on the serum creatinine-based diagnosis of AKI. Every morning, users of EHIS can see the automatic diagnosis of AKI and differential diagnosis of causes for AKI. 

As for the baseline serum creatinine value, we set the baseline value as follows: the lowest value within 2 days, the lowest value within 7 days, or the latest value within 6 months. First, the EWS screened the serum creatinine value that had been obtained in the last 2 days, and the latest value had to be higher by 0.3 mg/dL than the baseline serum creatinine value based on the KDIGO guidelines. If the value of serum creatinine was not obtained in the last 2 days, EWS screened the serum creatinine value that had been obtained in the last 7 days, and the latest serum creatinine value had to be 50% higher than the baseline serum creatinine value (using the lowest value as the baseline). Finally, if the required value had not been obtained in the last 7 days, but it had been obtained in the last 6 months, the latest value had to be 50% higher than the baseline value (using the latest value within 6 months as the baseline). 

### 2.2. Definition of AKI and Outcome Measures

We adopted the KDIGO definition of AKI [7,12]. Once diagnosed as AKI, we evaluated the outcome of AKI as AKD, CKD, and CKD G5 (dialysis-dependent). The term “AKD” defined by KDIGO [4,13] is defined as showing kidney damage for <3 months (structural criteria), with AKI or GFR < 60 mL/min per 1.732 m^2^ for <3 months or decreased in GFR by ≥35% or increased in serum creatinine by >50% for <3 months (functional criteria). CKD is defined as showing elevated serum levels of creatinine or GFR < 60 mL/min per 1.732 m^2^ for >3 months (functional criteria) or other evidence of kidney damage, with a presence >3 months (structural criteria) [5]. Those with dialysis dependence 3 months after AKI diagnosis were defined as CKD G5. 

We collected data on AKI incidence from September 2018 to January 2021. The trend line of daily incidence was separated in March 2020 (on board of EWS over EHIS). We defined daily AKI incidence rates (%) according to two definitions: case numbers of AKI/inpatients with data of serum creatinine (Definition 1) and case numbers of AKI/all inpatient with or without data of serum creatinine (Definition 2).

### 2.3. Endpoints

For daily monitoring of AKI incidence, we plotted the data in terms of a trend line, together with the equation and the coefficient of determination. We presented the mean incidence of AKI before and after EWS. As the daily incidence of AKI was not distributed normally, we also compared the different proportions of AKI incidence before and after EWS to assess the benefits of AKI-EWS. For outcome analyses (including full recovery, AKD, CKD, CKD G5, and mortality), we collected patients with AKI from September 2018 to November 2020. We also recorded the case numbers of consultation of nephrologists before and after the AKI-EWS for the analysis of clinical loading of nephrology. 

### 2.4. Statistical Analysis

Data are expressed as means, standard deviations, or percentages. Differences between incidence rates of AKI before and after notification were evaluated by Student’s *t*-test. A linear regression model was plotted for trend lines to visualize the trend of AKI incidence and the outcomes before and after EWS. A statistically significant difference was set at *p* < 0.05. All analyses were performed with the SPSS statistical software, version 12.0 (SPSS Inc., Chicago, IL, USA). All methods were carried out in accordance with human research guidelines and regulations of TCVGH (Institutional Review Board number: CW 17045A).

## 3. Results

### 3.1. Enlisting a Stakeholder Mapping

**Figure 1:**
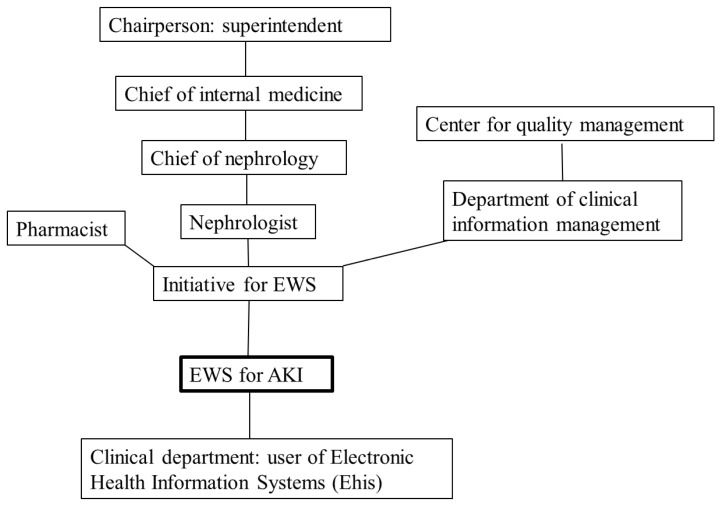
Stakeholder map for real-time early alert system of acute kidney injury.

First, we enlisted a stakeholder map for this QI project (Figure 1). The EWS had maximal applicability to relevant stakeholders. Its chairperson was the hospital superintendent, together with heads of internal medicine and nephrology. The Center for Quality Management and pharmacists were also recruited into this team. After creating the EWS for AKI, users from all clinical departments for EHIS were recruited (including all nurse practitioners, interns, residents, and visiting staff).

### 3.2. Action Plan of This Initiative

**Figure 2:**
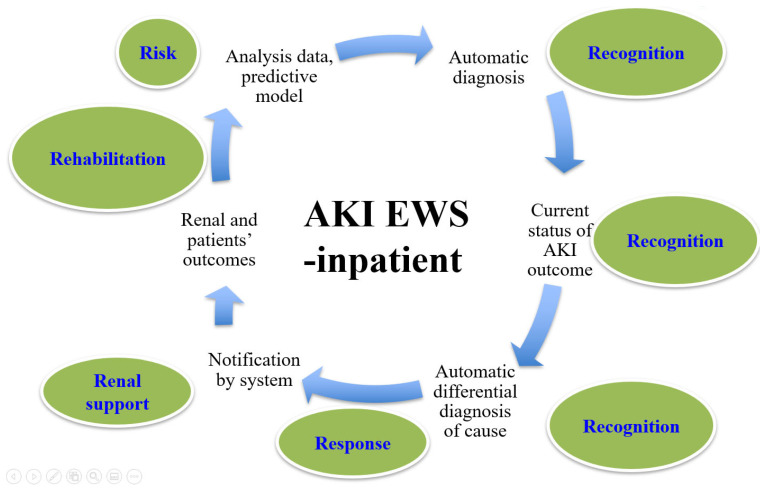
Action plan for acute kidney injury.

We then started an action plan for this initiative (Figure 2). First, the system had to be automatic for diagnosing AKI and had to increase awareness and recognition of AKI. Moreover, this system also provided real-time information on the daily incidence of AKI and AKI outcome status. Apart from automatic diagnosis of AKI, we also created automatic differential diagnosis on causes of AKI. We were able to then display the diagnosis of AKI, its disease stage, and possible causes to clinical users on our EHIS platform. Users were able to deal with AKI themselves or to consult nephrologists. In this system, we were able to check on patient outcomes, such as AKI, CKD, CKD G5, or mortality. All these data were fed back to our AKI-EWS core for system improvement.

### 3.3. Early Warning System (EWS) for Acute Kidney Injury (AKI) in the Electronic Health Information System (EHIS)

**Figure 3:**
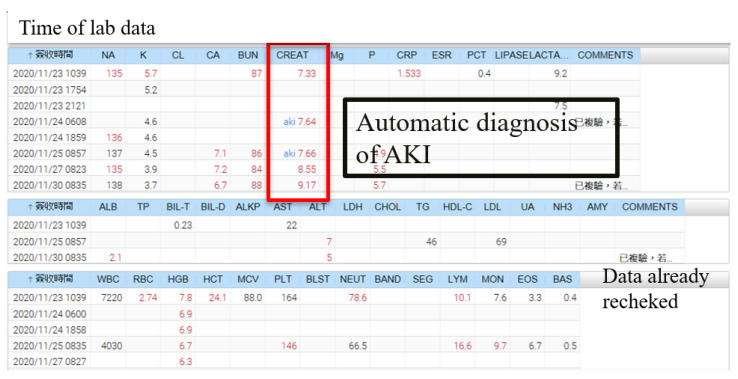
Real-time notification of acute kidney injury in electronic health information systems.

Next, this system was built into our inpatient EHIS (Figure 3). For a given patient, when the elevated creatinine level was compatible with the diagnosis of AKI according to KDIGO [7,12], a label “aki” was shown in the laboratory report. This was an automatic diagnosis of AKI. Clinical users thereby did not need to memorize criteria of AKI diagnosis [7,12], which were often too complicated to recall. In this AKI-EWS, clinical users were able to readily recognize their patients with AKI. In addition to the automatic diagnosis of AKI, clinical users were able to mouse-click on the label “aki” to show details of AKI causes with the automatic differential diagnosis (Supplementary Figure S1). In the example, this patient showed positive (which were marked by red asterisks) for anemia and non-steroidal anti-inflammatory drug (NSAID) (10 days before the episode of AKI) in the list of all possible causes of AKI, and negative for others (marked by green asterisks). In other cases, some causes of AKI (such as the severity of anemia) were not clearly defined. Clinical users were able to set an individualized threshold for anemia, after clicking on the “pen” icon in the right column (Supplementary Figure S2). All differential diagnoses of AKI causes were screened for all patients with AKI according to 3 types of AKI, including pre-renal, intrinsic, and post-renal type (Supplementary Figure S3). This automatic differential diagnosis of AKI causes was reported in current literature reviews. 

### 3.4. The Daily Incidence of AKI and the Effect of Intervention of AKI-EWS 

All adult inpatients (>20 y/o) without dialysis were included in this EWS without any exclusion. During the whole study period (before and after this intervention), the mean daily case numbers of all inpatients with creatinine data were 283.9 ± 117.8 and 291.6 ± 128.0 without statistical significance (*p* = 0.552). The mean daily case numbers of all inpatients with or without creatinine data were 1268.3 ± 150.8 and 1339.6 ± 101.4 with statistical significance (*p* < 0.001). The mean daily case numbers of all inpatients with AKI were 10.7 ± 3.6 and 11.1 ± 4.4 before and after this intervention without statistical significance (*p* = 0.286). 

**Figure 4:**
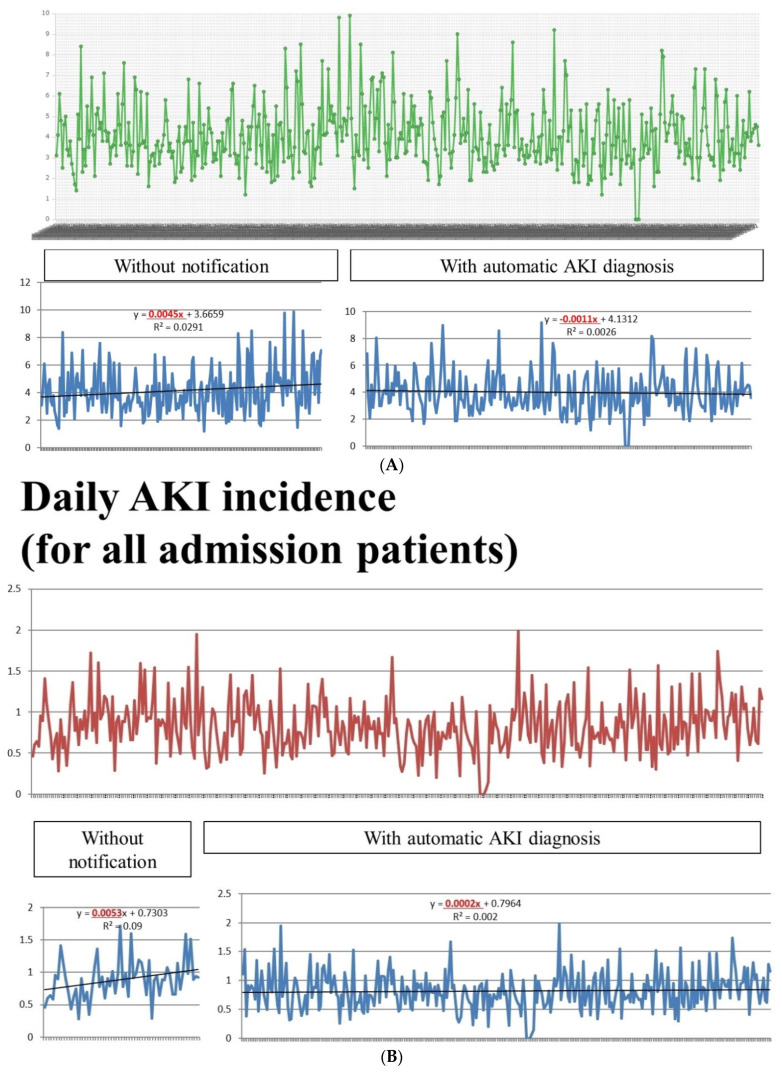
(**A**) Daily incidence (%) of acute kidney injury for patients with collected serum creatinine data before and after notification (Definition 1); (**B**) daily incidence (%) of acute kidney injury for admission patients before and after notification (Definition 2).

The daily incidence of AKI for admitted patients with data of serum creatinine (Figure 4A) (numerator/denominator = patients with AKI/ inpatients with data of serum creatinine) or for all admitted patients (with or without data of serum creatinine) was shown in this system (Figure 4B) (numerator/denominator = patients with AKI/all inpatients with our without data of serum creatinine). With this, we were able to monitor the incidence of AKI on a daily basis. The incidence of AKI for patients with laboratory data on serum creatinine ranged from 0 to 10% (Figure 4A) or from 0 to 2% for all admitted patients (Figure 4B). 

The incidence of AKI for inpatients with laboratory data on serum creatinine showed a declining trend (Figure 4A). The trend lines for AKI before and after notification were, respectively, as follows: y = 0.0045x + 3.6659 (R^2^ = 0.0291) and y = 0.0011x + 4.1312 (R^2^ = 0.0026). The trend (slope) for the daily incidence of AKI showed decreasing before and after the implementation of AKI-EWS (0.0045 vs. −0.0011). Before AKI-EWS, the daily incidence of AKI increased (positive slope, 0.0045). However, after the implementation of AKI-EWS, the daily AKI incidence began to decrease (negative slope, −0.0011). Similarly, the incidence of AKI for all inpatients with or without laboratory data on serum creatinine also declined (Figure 4B). The trend lines for AKI before and after notification were, respectively, as follows: y = 0.0053x + 0.7303 (R^2^ = 0.09) and y = 0.0002x + 0.7964 (R^2^ = 0.002). The slope for the daily incidence of AKI showed decreasing before and after the implementation of AKI-EWS (0.0053 vs. 0.0001). Based on the above findings, the daily incidence of AKI showed less decrease (Definition 1) or less increment (Definition 2). 

Table 1 shows the mean daily incidence of AKI before and after AKI-ESW. The mean incidence of AKI (for inpatients with data on serum creatinine) before the notification was 4.14% and after notification was 3.99% without a statistically significant difference (*p* = 0.40). Similarly, the mean incidence of AKI (for all inpatients with or without data on serum creatinine) before the notification was 0.89% and after notification was 0.82%, without a statistically significant difference (*p* = 0.085). We further compared various proportions of AKI incidence (i.e., >4%, >6%, >7%, and >8%) before and after AKI-ESW. We found the proportion of AKI > 4% was reduced significantly (47.7% and 41.6%, *p* = 0.010) in inpatients with data on serum creatinine. The proportion of AKI > 0.9% in all inpatients with or without data on serum creatinine was also reduced significantly (51.67% and 35.94%, *p* = 0.024).

### 3.5. Long-Term Outcomes of AKI before and after AKI-EWS 

**Figure 5:**
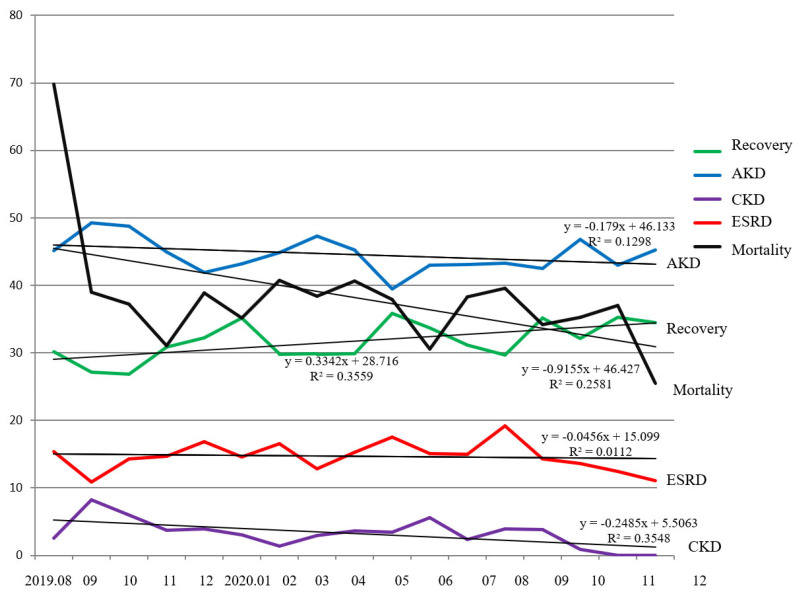
Outcomes of acute kidney injury.

Long-term outcomes of AKI during the whole period of our study are shown in Figure 5. The equation and the coefficient of determination of trend line for all outcomes of AKI before and after notification are as follows: y = 0.7276x + 27.397 (R^2^ = 0.2977) vs. y = 0.3846x + 30.6 (R^2^ = 0.2244) for recovery (*p* = 0.315); y = −0.7022x + 48.237 (R^2^ = 0.3195) vs. y = −0.0999x + 44.241(R^2^ = 0.0131) for AKD (*p* = 0.366); y = −0.5786x + 6.4484 (R^2^ = 0.2955) vs. y = −0.4093x + 4.9159 (R^2^ = 0.4547) for CKD (*p* = 0.087); y = 0.4808x + 12.846 (R^2^ = 0.2808) vs. y = −0.3307x + 16.452 (R^2^ = 0.172) for dialysis dependent (*p* = 0.092); y = −3.3312x + 55.02 (R^2^ = 0.3156) vs. y = −0.8671x + 40.501 (R^2^ = 0.3193) for mortality (*p* = 0.688). The intervention of AKI-EWS did not show a statistically significant improvement in long-term outcome. However, the trend lines of long-term outcomes showed improving tendency (positive slope for recovery (+0.3342), and negative slope for AKI (−0.1790), mortality (−0.9155), ESRD (−0.0456), and CKD (−0.2485)). The trend line (green line) of recovery from AKI showed an increased tendency: y = 0.3342x + 28.716 (R^2^ = 0.3559). For AKD (blue line), it also showed a decreasing tendency: y = −0.179x + 46.133 (R^2^ = 0.1298). Worse outcomes of AKI (including CKD—purple line, CKD G5—red line, and mortality—black line) also showed a decreasing trend: y = −0.2485x + 5.5063 (R^2^ = 0.3548) for CKD; y = −0.0456x + 15.099 (R^2^ = 0.0112) for dialysis dependent; y = −0.9155x + 46.427 (R^2^ = 0.2581) for mortality. 

### 3.6. Cass Numbers of Consultation of Nephrologists

**Figure 6:**
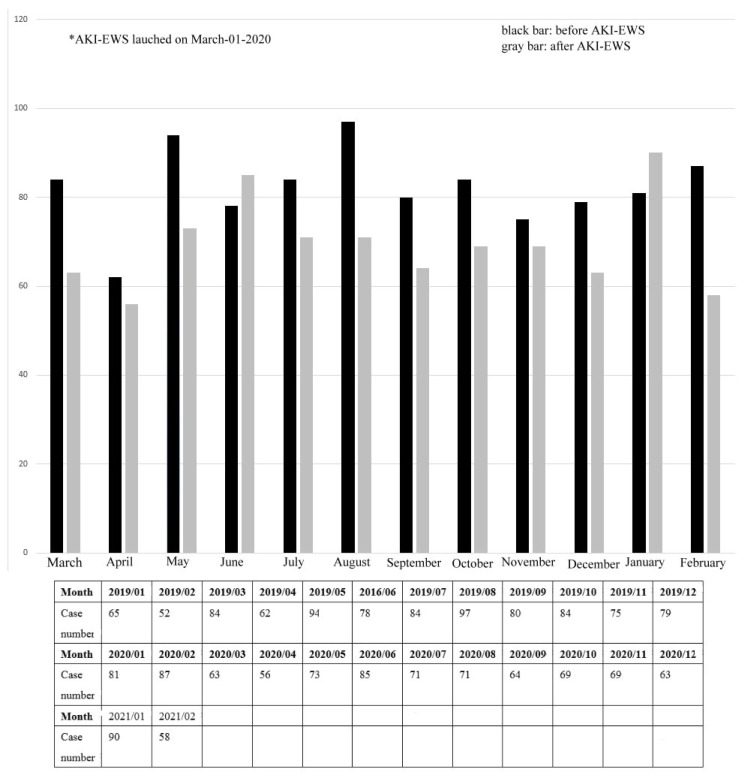
Case numbers of consultations of nephrologists before and after AKI-EWS.

On 1 March 2020, the AKI-EWS launched on our EHIS. Monthly case numbers of consultations of nephrologists are shown in Figure 6. All cases numbers of consultations one year before and after AKI-EWS nearly decreased except 2019/06 vs. 2020/06 and 2020/01 vs. 2021/01. One-year cases number of consultations before and after AKI-EWS were 985 and 832. The case number decreased by 15.5%. 

## 4. Discussion

AKI is common in hospitals, accounting for 13–18% of patients [14] and up to 60% of patients in the intensive care unit [15]. AKI is a “syndrome” with complex causes and mechanisms. AKI encompasses all conditions with a sudden loss of excretory renal function and is not considered as a specific disease, and without a specific mechanism of injury [16,17]. Automated and early alerting systems have a major influence on clinicians’ decision-making. A majority of hospitalized patients in this study were eligible for preventive measures, and computerized reminders significantly promoted the delivery of preventive measures to avoid complications [17]. However, there is no consensus regarding the benefits of AKI-EWS on patient outcomes. AKI might benefit from an individual with timely and early intervention. According to a systematic review [11], EWS is heterogeneous in design, variably implemented, and is rarely used for decision support. In a recent review [18], key elements to this approach are the delineation of AKI under the five Rs—risk assessment, recognition, response, renal support, and rehabilitation. In our study, after AKI-ESW implementation, the incidence of AKI dropped with statistical significance, and patient outcomes improved (more recovery, and less CKD, CKD G5, and mortality). Here, we highlight the unique characteristics of our AKI-EWS according to 5Rs. 

First (risk assessment), identifying high-risk patients is pivotal for preventive strategy. The keystone of any intervention plan for AKI is that many episodes of AKI are preventable, are amenable to early detection, and are treatable [18]. For example, Cho et al. reported on a computerized alert program to alarm physicians and to recommend prophylactic measures in high-risk patients for contrast-related AKI. EWS lowers the incidence of contrast nephropathy (3% vs. 10%) [19]. That EWS lowered the incidence of contrast nephropathy (3% vs. 10%) [19]. In our AKI-EWS, we were able to set individual thresholds by all users to increase the sensitivity of AKI and to identify high-risk patients, such as those with creatinine increasing with time, hemoglobin dropping with time (Supplementary Figure S2), or blood pressure dropping with time. Low hematocrit is associated with AKI development [20,21,22,23]. However, no evidence-based data are available on the cut-off value of hemoglobin or the speed of hemoglobin decline. We were able to set an individualized threshold to identify patients with high risk. With high-risk subjects identified, the assessment of AKI risks was performed by nephrologists as well as by other related specialists. The tailored and individual threshold setting by users is a key point of our AKI-EWS.

Second (recognition), the key concept for timely treatment of AKI is the prompt diagnosis of AKI to avoid further insult and progression of the renal condition [24,25]. In our AKI-EWS, we had a short time window during which AKI was diagnosed on this rule-based system because we screened laboratory reports on serum creatinine for all inpatients on a daily basis. Additionally, the automatic diagnosis of AKI facilitated the diagnosis by unloading physicians from the complicated AKI criteria. For most other AKI-EWS, timely recognition of AKI is their major benefit. 

Third (response and renal support), the response to AKI from clinicians is also pivotal for patient outcomes. The response to EWS depends on the contents of EWS and the integration of clinical decision support, both of which vary according to different EWS. In our EWS, we targeted all EHIS users by showing the diagnosis of AKI on the page of laboratory data. When users of EHIS viewed their patients’ data, they were able to see the AKI alarm message in real time. We did not choose e-mail [26] or electronic medical record (EMR) [27,28] which would have caused delays in notification. To avoid alert fatigue, we also did not choose the intrusive e-alert approach (such as text messages to physicians’ mobile phones [29]). In our opinion, our e-alert system is between the passive and the intrusive approaches and hence obtained both benefits. 

In addition to the users’ response, renal support also mattered. Automatic nephrologist consultation is common in hospital AKI-EWS [27,30]. Once detecting AKI, there is automated nephrologist consultation according to a before-and-after quality improvement study [30]. After introducing EWS and automated nephrologist consultation, the odds of overlooking AKI cases were significantly reduced (adjusted OR, 0.40; 95% CI, 0.30–0.52) [30]. The outcomes of AKI improved in that study [30]—namely, reduced odds of severe AKI (adjusted OR, 0.75; 95% CI, 0.64–0.89) and improved likelihood of AKI recovery (adjusted HR, 1.70; 95% CI, 1.53–1.88). Therefore, the response of AKI really matters. However, considering the volume of patients in our institute, we chose other responses to AKI-EWS before consulting a nephrologist. We created a system with an automatic diagnosis on the “cause” of AKI, which had never been reported in the literature (Supplementary Figure S3). The system screened all possible “causes” of AKI in <5 s, and results were shown in our EHIS. Without consultation with a nephrologist, clinicians can still have renal support from this AKI-EWS system. A straightforward message on AKI detection and its cause was sent to the AKI health providers. This real-time and ready-to-use system detecting AKI and its cause also helped to avoid the disease progression. The time taken for automatic detection of the AKI cause was shorter than that through consulting the nephrologist. The loading of consultation of nephrologists decreased 15.5% after the AKI-EWS. Until now, this system is the fastest AKI-EWS in automatic detection of the AKI causes. This feature is a key element of our AKI-EWS in reducing daily AKI incidence and improving patient outcomes. 

Based on the above reasons, our AKI-EWS was well designed and ready to use. Moreover, this proposed AKI-EWS was for all users of EHIS and was a hospital-wide system to cover all inpatients in our institute. As is well known, AKI is usually first encountered by nonspecialized healthcare providers. Only 10–15% of AKI patients were seen by nephrologists [31,32]. Non-nephrologists might not have the training for early recognition and timely intervention. Apart from diagnosing AKI, this AKI-EWS was also an education system with a self-differential diagnosis on the causes of AKI. 

There are some limitations of our study. First, our EWS did not include the parameter of urine amount. However, in clinical practice, it is a real clinical scenario of daily life. We plan tocreat a system that can be used for real-world situations in daily clinical practice. Second, our intervention of AKI appeared somewhat insufficiently strong. However, this was meant to be a preliminary study, aiming to establish a diagnostic system first. Our system had a novel function in screening the cause of AKI automatically. Clinicians can study the cause of AKI and manage it as soon as possible by themselves. In other words, this system will not overload our nephrologists. Third, we cannot prove the causal relationship between EWS and better outcomes of AKI, despite its help to clinicians in the early diagnosis of AKI and early intervention. In the future, we will perform a subgroup study with objective causal effects on AKI, such as contrast, shock, and NSAID. We believe this can help clinicians to reduce the incidence of AKI and prevent AKI-related comorbidity. Fourth, there are various confounding factors associated with the outcomes of AKI. We did not have these data in this study. Finally, we will study more preventable and treatable diseases related to AKI in the future. Our system hopefully can timely identify patients at risk of AKI for preventive measures.

## 5. Conclusions

By implementing a well-designed AKI-EWS, the incidence of AKI was reduced, and its outcome improved with reduced loading of consultation with a nephrologist. Our AKI-EWS’s benefits depended on its high-risk identification (individual threshold detection), timely and automatic diagnosis, real-time alerting on EHIS, quickly self-diagnosing the cause of AKI, and coverage of all inpatients.

## Figures and Tables

**Table 1:** Daily AKI (for all patients with creatinine data and all admissions with or without creatinine).

	Before Notification	After Notification	*p* Value
Mean daily case numbers of all inpatients with AKI (n ± standard deviation)	10.7 ± 3.6	11.1 ± 4.4	0.286
Definition 1: Daily AKI incidence (%) (for inpatients with creatinine data)			
Mean daily case numbers of all inpatients with creatinine data (n ± standard deviation)	283.9 ± 117.8	291.6 ± 128.0	0.552
Mean incidence of AKI	4.14%	3.99%	0.4
Proportion of AKI > 4%	47.4%	41.6%	**0.010**
Proportion of AKI > 6%	15.02%	11.02%	0.440
Proportion of AKI > 7%	5.63%	4.16%	0.590
Proportion of AKI > 8%	2.82%	2.08%	0.973
Definition 2: Daily AKI incidence (%) (for all inpatients with or without creatinine data)			
Mean daily case numbers of all inpatients with or without creatinine data (n ± standard deviation)	1268.3 ± 150.8	1339.6 ± 101.4	<0.001
Mean incidence of AKI	0.89%	0.82%	0.085
Proportion of AKI > 0.8%	58.33%	49.67%	0.221
Proportion of AKI > 0.9%	51.67%	35.94%	**0.024**
Proportion of AKI >1.0%	25%	24.51%	0.936

AKI: acute kidney injury.

## Data Availability

No additional data available.

## Supplementary Materials

The following supporting information can be downloaded at: https://www.mdpi.com/article/10.3390/ijerph19063704/s1, Figure S1:Automatic differential identify cause of acute kidney injury, Figure S2: Function of adjustment of threshold by users, Figure S3: All screening causes of acute kidney injury.
