# Supplementary material for: An Early Warning System for the Differential Diagnosis of In-Hospital Acute Kidney Injury for Better Patient Outcome: Study of a Quality Improvement Initiative
Source: Int J Environ Res Public Health. 2022 Mar 20;19(6):3704. doi: 10.3390/ijerph19063704 (PMC8953354; doi:10.3390/ijerph19063704)

Supplementary data

Figure S1. Automatic differential identify cause of acute kidney injury.

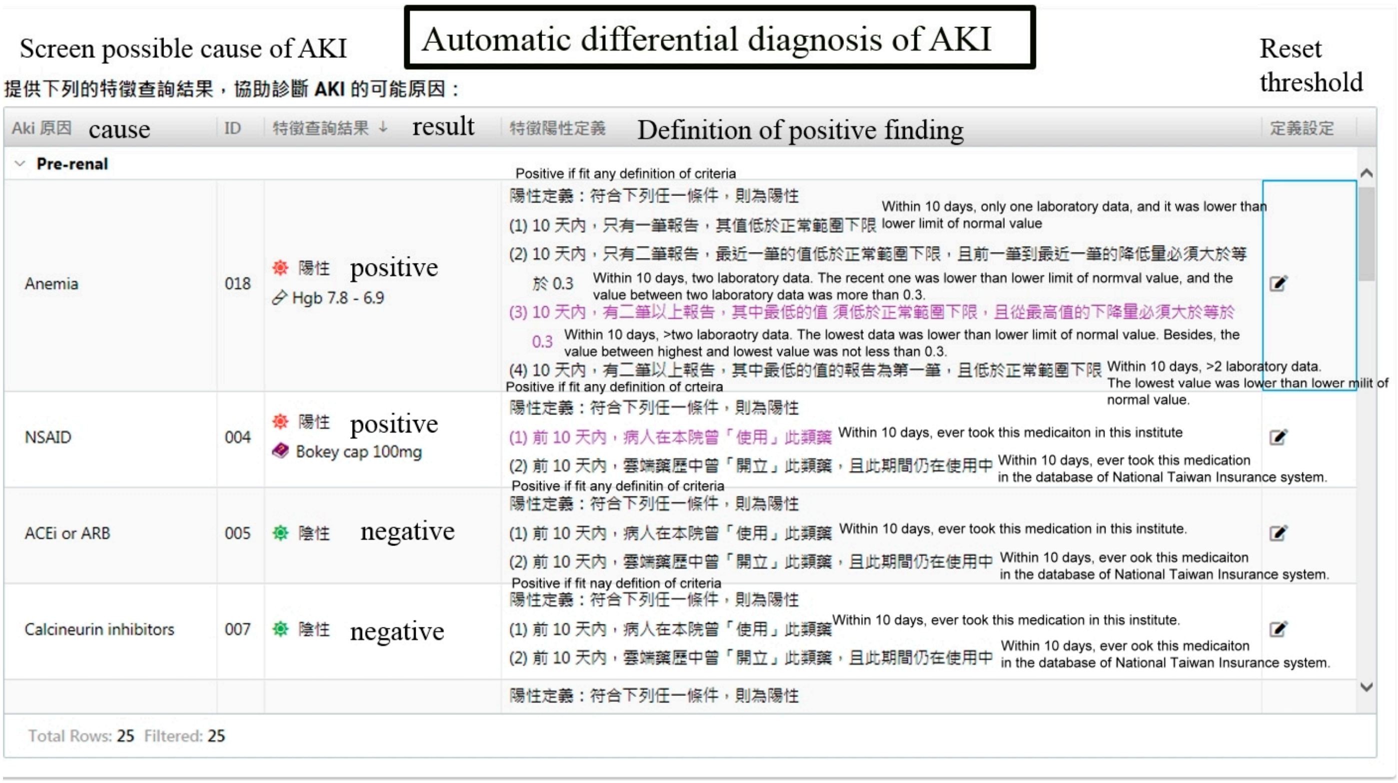

Figure S2. Function of adjustment of threshold by users.

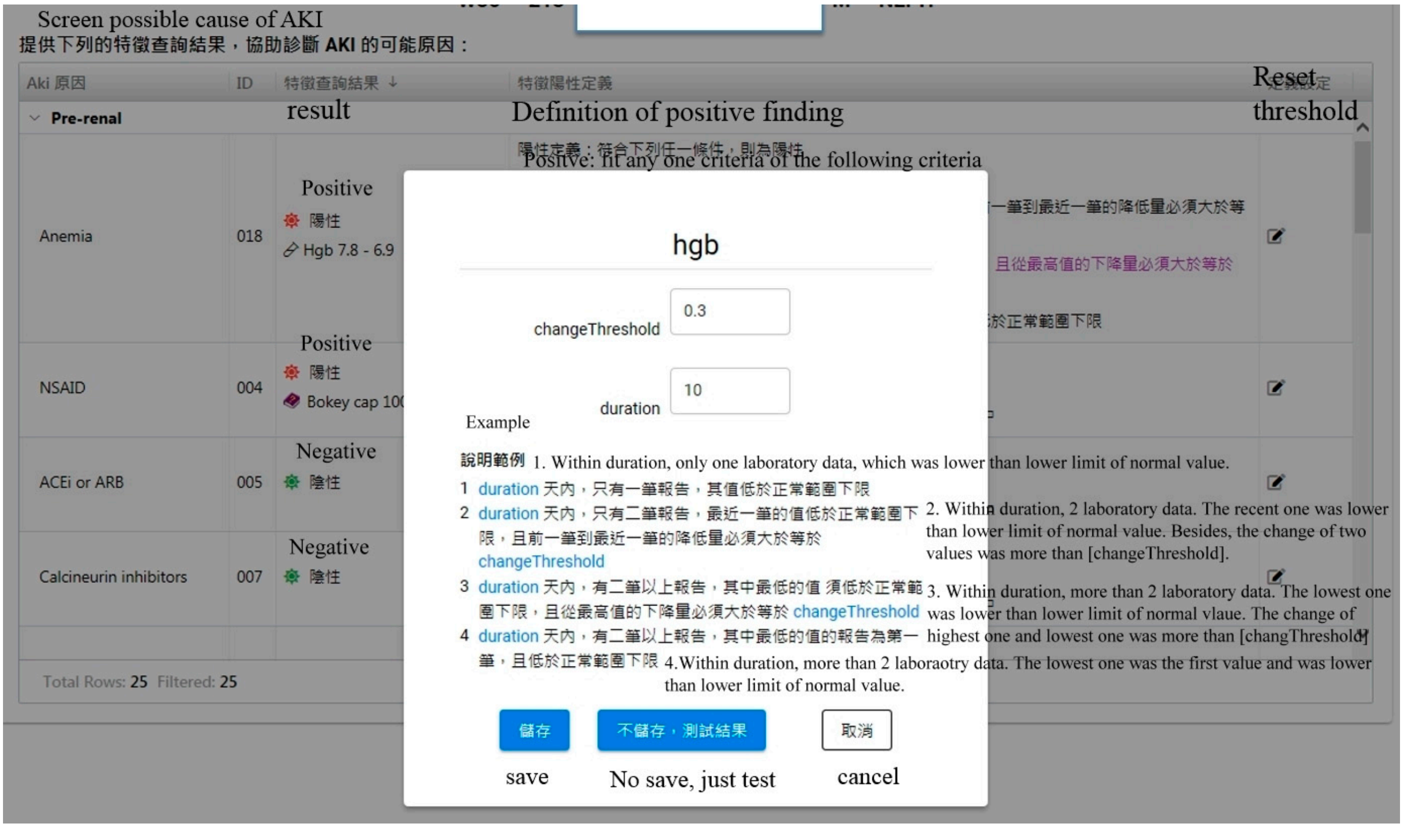

Figure S3. All screening causes of acute kidney injury.

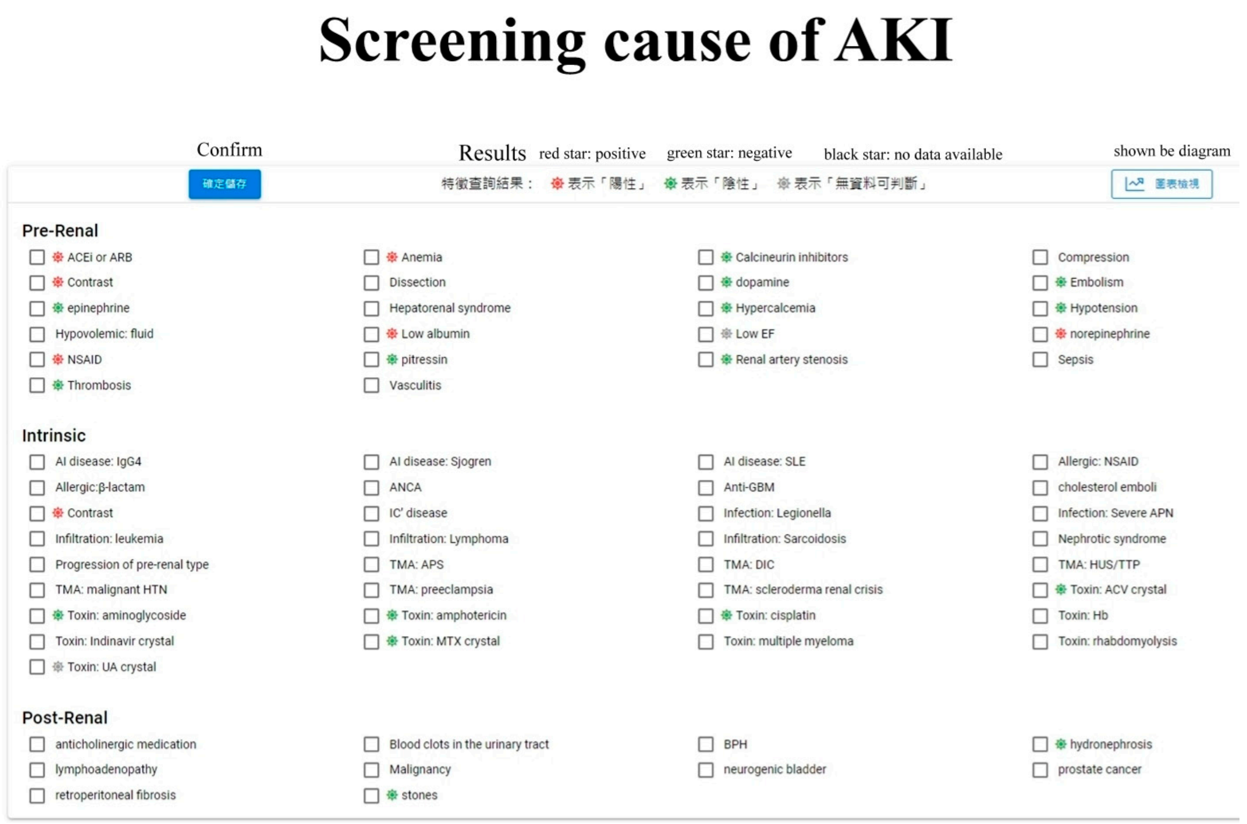

Supplement: Supplementary file 1 [file ijerph-19-03704-s001.zip › ijerph-1573924-supplementary.pdf]
